# A Historical Overview of Research on *Babesia orientalis*, a Protozoan Parasite Infecting Water Buffalo

**DOI:** 10.3389/fmicb.2017.01323

**Published:** 2017-07-14

**Authors:** Lan He, Qin Liu, Baoan Yao, Yanqin Zhou, Min Hu, Rui Fang, Junlong Zhao

**Affiliations:** ^1^State Key Laboratory of Agricultural Microbiology, College of Veterinary Medicine, Huazhong Agricultural University Wuhan, China; ^2^Key Laboratory of Development of Veterinary Diagnostic Products, Ministry of Agriculture, Huazhong Agricultural University Wuhan, China; ^3^National Institute of Parasitic Diseases, Chinese Center for Disease Control and Prevention, Key Laboratory of Parasite and Vector Biology, Ministry of Health, WHO Collaborating Center for Tropical Diseases Shanghai, China

**Keywords:** *Babesia orientalis*, babesiosis, water buffalo, emerging diseases, P. R. China

## Abstract

*Babesiosis* is a globally important zoonotic disease caused by tick-borne intraerythrocytic protozoan of the genus *Babesia* (phylum apicomplexa). In China, there are five species that infect cattle buffalo and cause great economic loss, which include *Babesia bigemina*, *B. bovis*, *B. major*, *B. ovata*, and *B. orientalis*. Among them, *B. orientalis* is the most recently identified new *Babesia* species epidemic in China. This review summarized the work done in the past 33 years to give an overview of what learned about this parasite. This parasitic protozoan was found in 1984 in Central and South China and then named as *B. orientalis* in 1997 based on its differences in transmitting host, morphology, pathogenicity and characteristics of *in vitro* cultivation when compared with *B. bigemina* and *B. bovis*. It was found that *Rhipicephalus haemaphysaloides* is the transmitting vector and water buffalo is the only reported host. Phylogenetic analysis based on the 18S rRNA gene also confirmed that *B. orientalis* is a new species. After species verification, four diagnostic methods including semi-nest PCR, loop-mediated isothermal amplification assay, reverse line blot hybridization assay, and real-time PCR were established for lab and field use purposes. Genomic sequencing was conducted and the complete genomes of mitochondria and apicoplast were annotated. Future work will be focused on developing effective vaccines, identifying drug targets and screening useful drugs for controlling *B. orientalis* in water buffalo.

## Introduction

**FIGURE 1:**
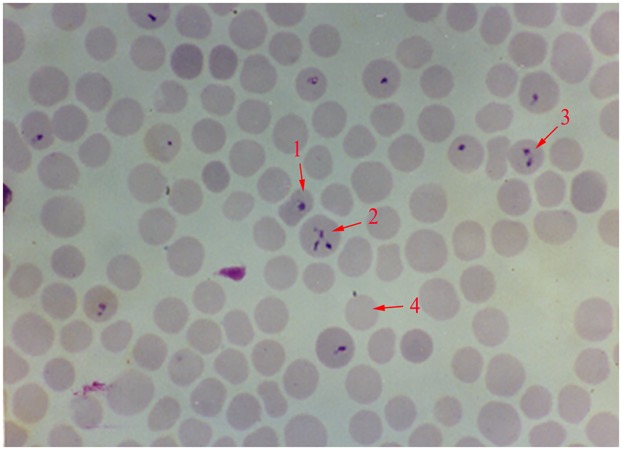
Giemsa stained thin blood smear of *Babesia orientalis* in water buffalo erythrocytes. Final magnification is 1000×. 1, single pyriform; 2, multiple parasites infected RBC; 3, double pyriform; 4, exo-erythrocytic merozoites.

*Babesia orientalis* is a tick-borne apicomplexan parasite infecting red blood cells and causing water buffalo babesiosis. It is one of the most important diseases of water buffalo in central and south China, resulting in huge economy loss annually (Chen et al., 1984, 1988). The clinical manifestations of this disease include fever, anemia, icterus, hemoglobinuria and even death (Zhang, 1984). In the beginning, *B. orientalis* was considered as *B. bovis* or *B. bigemina* due to their similar shape when it was found in 1984 in Hubei province, China (Chen et al., 1984, 1989). The parasite was identified as a new species and named *B. orientalis* in 1997, according to the differences in transmitting vector, morphology, pathogenicity and characteristics of *in vitro* cultivation when compared with *B. bovis* and *B. bigemina* (Ma et al., 1989; Zhao et al., 1997). Water buffalo has been identified as the only natural host and *Rhipicephalus haemaphysaloides* is the only vector for *B. orientalis* (Ma et al., 1989). The vector transovarially transmits the parasite. Infected females can transmit *B. orientalis* to their offspring. Compared with *B. orientalis*, *B. bovis* and *B. bigemina* are transmitted by both *Rhipicephalus* and *Ixodes* to cattle and buffalo (Liu et al., 2005; Uilenberg, 2006). As a member of Babesiidae family, *B. orientalis* has a sexual stage within *R. haemaphysaloides*, followed by an asexual stage in water buffalo erythrocytes (**Figure 1**). Phylogenetic analysis based on the nuclear 18S rRNA genes, the amino acid sequences of mitochondrial *cox1* and *cob* genes and heat shock protein 70, confirmed that *B. orientalis* is a new species which is distinct from *B. bigemina* and *B. bovis* (Liu et al., 2005; He et al., 2009a, 2014). After verification of the new species status of *B*. *orientalis*, series work has been done in order to maintain the parasites *in vitro*, establish diagnostic methods for use in the lab and field, understand the epidemiology and transmission patterns for design and implement of control strategies and to obtain genome sequence information for future drug discovery and vaccine development.

## Distribution Of *B. orientalis*

**FIGURE 2:**
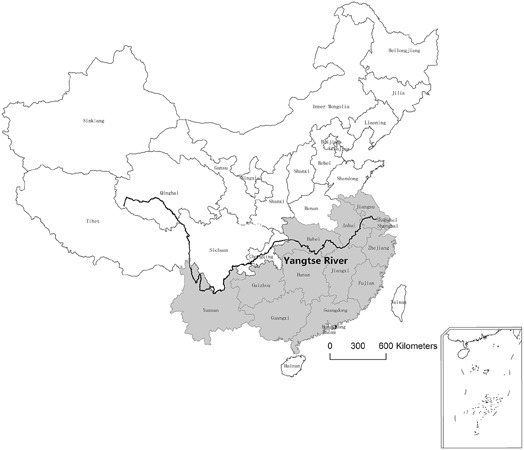
Distribution of *B. orientalis* in water buffalo in China. Black line, Yangtze River; gray, provinces which have been reported *B. orientalis* positive are marked with gray.

The epidemiology of babesiosis is usually related to the activities and distribution of tick vector. The prevalence of transmitting vector *R. haemaphysaloides* was reported from April to October, with peak period in May, June, and July. In the beginning, when using microscopic methods for diagnosis, *B. orientalis* was found only prevalent south of Yangtze River, including provinces of Hubei, Anhui, Jiangsu, Zhejiang, Jiangxi, Hunan, Guizhou, Yunnan, Guangxi, Guangdong, and Fujian (Yao et al., 2002). In 2007, an epidemiological investigation using semi-nested PCR detected samples from Hubei province. The results confirmed that *B. orientalis* was only prevalent to the south of Yangtze River, possibly due to the geographical reasons and the tick’s distribution. The river was considered as a natural barrier. The areas Jiayu, Wuhan, Anshan, and Daye of Hubei province which were south of the river were endemic areas. The counties north of Yangtze River side, including Macheng, Xiaogan and Hongan of Hubei province were *B. orientalis* free (Liu et al., 2007). However, in a later study, 1 (1/88) and 2 (2/88) positive cases were reported north of Yangtze River by semi-nest PCR and a loop-mediated isothermal amplification assay (LAMP), respectively (He et al., 2009b). The finding means *B. orientalis* had been spread to non-epidemic areas (**Figure 2**), possibly by convenient transportations of water buffalo from south to north because more and more bridges built on the river make the river no longer a natural barrier. In 2011, 14% (16/114) field samples collected north of Yangtze River were positive by real-time PCR (He et al., 2011). The results further confirmed that *B. orientalis* was spread rapidly and the natural barrier was destroyed. This is a serious threat to the water buffalo industry (He et al., 2009b).

## *In Vitro* Cultivation

*In vitro* cultivation of *B. orientalis* was conducted in 1991 (Zhao and Liu, 1991). The media used (pH 7.2) consisted of M199 medium (Gibco, United States) with 40% adult water buffalo serum. *B. orientalis* was cultivated in 24-well plate at 37°C with a gas mixture of 2% oxygen, 5% carbon dioxide, and 93% nitrogen (MASP) (Zhao and Liu, 1991; Zhao et al., 1994). The parasites were split every 2 or 3 days depends on the parasitemia and could be continuously cultured for 26 generations in 80 days. The cultured parasitemia at 24, 48, and 72 h were 2.63 ± 0.50%, 7.18 ± 1.39% and 20.78 ± 4.52%, respectively, and the highest percentage of parasitemia (PPE) could reach 33.50% (Zhao and Liu, 1991; Zhao et al., 1997). For setting up the *in vitro* culture, infected water buffalo blood (donor of *B. orientalis*) and health water buffalo RBCs (donor of RBC) were needed. According to the record, infected water buffalo blood could be stored for 20 days in 4°C, the parasites still alive for setting up cultivation. Whereas health water buffalo RBC could be only stored for 6 days in 4°C before use (Zhao et al., 1998a,b).

## Detection

Major deterrents to diagnosis of babesiosis include low index of suspicion and non-specific clinic manifestations. Microscopical inspection of blood smear is a traditional diagnostic method for piroplasma. However, it remains challenging for monitoring carrier and recovered animals, and difficult to differentiate species of *Babesia* and *Theileria*. Thus, low sensitivity and specificity is one of the most important reasons for developing other testing method. Since 2002, useful serological and molecular methods have been developed for *B. orientalis*. The first and only serological method was latex agglutination test (LAT) which was established based on *in vitro* culture excreted antigens of *B. orientalis* (Yao et al., 2002). A semi-nested PCR was developed targeting the 18S rRNA gene, to investigate the epidemiology and enzootic potential in 2007 (Liu et al., 2007). After that, a LAMP assay was established with high sensitivity and specificity. The LAMP assay was able to detect *B. orientalis* on 3 days post-infection whereas microscopy and semi-nested PCR could only detect the parasite on 9 and 6 days after infection, respectively. The results mean LAMP is capable for early diagnosis of the infection (He et al., 2009b). However, none of these tests can monitor the parasitemia of infected animals. A real-time PCR was then developed for the quantitative analysis of *B. orientais* in water buffalo in 2011 (He et al., 2011). As we know, there are more than one hundred *Babesia* species in the worldwide. At least five species, *B. bigemina*, *B. bovis*, *B. major*, *B. ovata*, and *B. orientalis*, has been identified in cattle and/or buffalo in China (Yin et al., 1997; He et al., 2012). It is difficult to discriminate the mixed infections which are very common in clinic cases. To solve this problem, a very practical assay, reverse line blot hybridization (RLB), reported by Gubbels and Georges, has been adapted to detect haemoparasites. It can simultaneously detect and differentiate 45 species of protozoan parasites in one test (Gubbels et al., 1999; Georges et al., 2001). In 2012, a specific probe was designed to test *B. orientalis* by RLB. The results indicated that RLB can effectively detect *B. orientalis* in field samples from both single and mixed infections (He et al., 2012). The reason for developing those detection methods is to fit different requirements. LAT is useful in detecting sub-clinical cases and field surveys. LAMP is low cost, simple, rapid with high specificity and efficiency, could be used for initial stages of infection. Real-time PCR is the only quantitative method of *B. orientalis*, can test the parasitemia of infected animals. RLB is normally used to distinguish mixed infections.

## Genome Sequencing

The first reported genome of *Babesia* was *B. bovis*, which has four chromosomes with a total size of 8.6 Mbp (Brayton et al., 2007). After that, genomes of a number of *Babesia* species were sequenced, including *B. divergence*, *B. bigemina*, *B. microti*, and *B.* sp. Xinjiang with sizes of 10.8, 13.8, 6.5, and 8.4 Mbp, respectively (Cornillot et al., 2012; Jackson et al., 2014; Guan et al., 2016). The genome sequencing of *B. orientalis* was started in 2009. The results showed that the parasite has four chromosomes. 1284 scaffolds were obtained after whole genome assembly, the total size was 7.8 Mbp, with a GC content of 41.8% (**Table 1**). The sequencing also demonstrated that *B. orientalis* harbors two extranuclear organelles, mitochondrion and a semi-autonomous plastid–like organelle named apicoplast, which is similar to the other members of apicomplexa parasites. The entire mitochondrial genome is a linear form with 5996 bp in length. It contains three protein-coding genes, *cox1*, *cob*, and *cox3*, in accordance with the mitochondrial genomes of other apicomplexa parasites. Multiple sequence alignment showed that *B. orientalis* mitochondrial genome is similar to that of the related apicomplexa parasites (He et al., 2014). The apicoplast genome was sequenced and annotated in 2015. It consists of a 33.2 kbp circular DNA with a high A+T content of 78.9% (Huang et al., 2015). Further analysis indicated that the apicoplast of *B. orientalis* contains a very important isoprenoid biosynthesis (MEP) pathway. There are seven enzymes work in MEP pathway, including DXS, DXR/IspC, IspD, IspE, IspF, IspG, and IspH, which is similar to previous reported apicomplexan MEP pathway (Imlay and Odom, 2014). It was reported that drugs, fosmidomycin targeting the biosynthesis of isopernoids caused rapid growth arrest and death of parasites, and fosmidomycin has been reported as a inhibitor targeting the second, speed limiting enzyme DOXP reductoisomerase (DXR) (Chakraborty, 2016). The genes encoding seven enzyme of MEP pathway were cloned, and the proteins have been identified in *B. orientalis.* Drug test assay showed that fosmidomycin could reduce the parasitemia of *in vitro* culture in 24 h (data not published). The results demonstrated that the existence of MEP pathway in *B. orientalis* and could be a potential drug target for controlling water buffalo babesiosis.

**Table 1:** Features of *Babesia orientalis* draft genome compared with that of *Plasmodium falciparum*, *Theileria parva*, and *B. bovis*.

Features	Species			
	*P. falciparum*	*T. parva*	*B. bovis*	*B. orientalis*
Size (Mbp)	22.8	8.3	8.2	7.8
Number of chromosomes	14	4	4	4
Total G+C composition (%)	19.4	34.1	41.8	42.4
Size of apicoplast genome (kbp)	35	39.5	33	33
Size of mitochondrial genome (kbp)	6 (linear)	6 (linear)	6 (linear)	6 (linear)
Number of nuclear protein coding genes	5,268	4,035	3,671	4199
Average protein coding gene length (bp)	2,283	1,407	1,514	1099
Percent genes with introns	53.9	73.6	61.5	46.1
Mean length of intergenic region (bp)	1,694	405	589	1100.0
G+C composition of intergenic region	13.8	26.2	37	36.2
G+C composition of exons (%)	23.7	37.6	44	44.59
G+C composition of introns (%)	13.6	25.4	35.9	35.8
Percent coding	52.6	68.4	70.2	61.2
Gene density	4,338	2,057	2,228	1856.7


## Control Strategies

Targets for controlling the transmission of *B. orientalis* include three elements, water buffalo, the vector *R. haemaphysaloides* and the parasites. Control the ticks will be the most efficient way for controlling tick-borne diseases. There are variety of chemicals can reduce the tick populations directly. However, it is impossible to eradicate all the ticks in nature. The other strategy is to control *B. orientalis* in water buffalo. It was reported that the secretory antigens derived from *in vitro* cultivation could induce protection to the virulent of *B. orientalis*, and could be used as a vaccine to prevent the disease (Zhao et al., 1997, 2002). Water buffalo in the vaccinated group exhibited a slight decrease in hemoglobin levels, blood cell counts. The control group showed typical clinical manifestation with fever, anemia haemoglobinuria and died between day 11 and 16 post-infection (Zhao et al., 2002). Another way for controlling *B. orientalis* is using drugs. There is no specific literature record for *B. orientalis* drug treatment. According to our experience in clinic, one dose of 4 mg/kg intramuscularly (IM) diminazene aceturate is the recommended treatment.

## Future Research

It has been more than 30 years since the parasite was discovered in water buffalo in Hubei province, China, and 20 years since it was named as *B. orientalis* in 1997. Many studies have been done, such as diagnostic methods, vaccine development, genome sequencing, and most importantly, *in vitro* cultivation was successfully established as it is the basic and core technique for *Babesia* research. However, little is known about the biology and pathogenesis of the parasite at molecular level. What is the molecular mechanism of *R. haemaphysaloides* transmits *B. orientalis* to water buffalo? How does *B. orientalis* interact with the host, invade red blood cells, grow and proliferate? Several antigens, such as AMA1, RAP1, BoP34, etc., have been identified and presumed to play important roles in *B. orientalis* invasion of RBC (Yu et al., 2014; He et al., 2015a,b). There is no report about the receptors and/or interacting proteins for these antigens. On the other hand, there is no commercial vaccine available for disease control. The only reported vaccine which contains secretion-excretion antigens derived from *in vitro* cultivated parasites, is expensive and difficult to obtain. Further research will be focused on the understanding of the invasion functions, developing effective vaccines, identifying drug targets and screening useful drugs for controlling *B. orientalis* in water buffalo, and also the biology and molecular mechanism of vector transmission.

## Author Contributions

LH wrote the draft of the manuscript. JZ and MH revised the manuscript. LH, YZ, MH, RF, and JZ are presently working in Parasitology lab, Huazhong Agricultural University, China. They contribute the basic need for *B. orientalis* research. QL was a Ph.D. student in the lab from 2001 to 2006. She did the first phylogenetic study and established semi-nested PCR. BY worked in the lab before he retired, he investigated the epidemic areas, studies life cycle and pathogenicity of *B. orientalis*. All authors read and approved the final manuscript.

## Conflict of Interest Statement

The authors declare that the research was conducted in the absence of any commercial or financial relationships that could be construed as a potential conflict of interest. The reviewer ME-G and handling Editor declared their shared affiliation, and the handling Editor states that the process nevertheless met the standards of a fair and objective review.
